# Association of Reproductive Factors and Postmenopausal Sex Hormones With Cognitive Impairment in Postmenopausal Women With Parkinson's Disease

**DOI:** 10.1002/cns.71182

**Published:** 2026-10-08

**Authors:** Wen‐Zheng Hu, Qin Wang, Ling Tang, Jia‐Qi Wang, Di Hu, Teng‐Teng Wu, Yue Huang, Zhi‐Hong Shi, Yi Ren, Zhi‐Rong Wan, Ping‐Yi Xu

**Affiliations:** ^1^ Department of Neurology The First Affiliated Hospital of Guangzhou Medical University Guangzhou China; ^2^ School of Nursing Guangzhou Xinhua University Guangzhou China; ^3^ Human Brain and Tissue Bank, China National Clinical Research Center for Neurological Diseases, Beijing Tiantan Hospital Capital Medical University Beijing China; ^4^ Centre for Healthy Brain Aging (CHeBA), Discipline of Psychiatry and Mental Health, School of Clinical Medicine, Faculty of Medicine and Health University of New South Wales Sydney New South Wales Australia; ^5^ Department of Neurology Huanhu Hospital Affiliated to Tianjin Medical University Tianjin China; ^6^ School of Basic Medical Sciences Guangzhou Medical University Guangzhou China; ^7^ Department of Neurology Aerospace Center Hospital Beijing China

**Keywords:** cognitive impairment, Parkinson's disease, postmenopausal women, predictive nomogram, reproductive factors, reproductive lifespan, sex hormones

## Abstract

**Aims:**

To explore the associations of reproductive factors (RFs) and postmenopausal sex hormone (PSH) with cognitive impairment (CI) in postmenopausal women with PD (PWPD), and to develop a predictive nomogram.

**Methods:**

Ninety‐five PWPD were divided into CI (*n* = 72; Montreal Cognitive Assessment [MoCA] < 26) and normal cognition (*n* = 23; MoCA ≥ 26) groups. RFs and serum PSH levels were collected. Risk factors were screened by logistic regression and confirmed via sensitivity and subgroup analyses. Model performance was evaluated using the area under the receiver operating characteristic curve (AUC), calibration curves, and decision curve analysis.

**Results:**

Shorter reproductive lifespan (RLS) and lower education independently predicted CI in PWPD. Compared with RLS ≥ 39 years, RLS ≤ 32 years (adjusted OR = 12.41, *p* = 0.011) and 33–38 years (adjusted OR = 8.42, *p* = 0.008) were associated with significantly higher CI risks. The RLS‐CI association remained stable across sensitivity analyses and was more prominent in milder‐stage patients. No intergroup PSH differences were observed. A nomogram integrating RLS, education, age, disease duration, and Hoehn and Yahr stage (H&Y stage) demonstrated satisfactory discrimination (AUC = 0.903), calibration, and net benefit.

**Conclusion:**

Shorter RLS independently predicts CI in PWPD, particularly in milder‐stage patients, while PSH shows no association. This nomogram may serve as a practical screening tool for CI in PWPD, pending external validation.

## Graphical abstract

Shorter reproductive lifespan constitutes an independent risk factor for cognitive impairment among postmenopausal women with Parkinson's disease, while postmenopausal sex‐hormone levels exhibit no relevant correlation with such cognitive impairment. We constructed a nomogram integrating reproductive lifespan, age, disease duration, education level, and Hoehn and Yahr stage (H&Y stage), which may serve as a promising screening tool for cognitive impairment in this population, pending external validation.

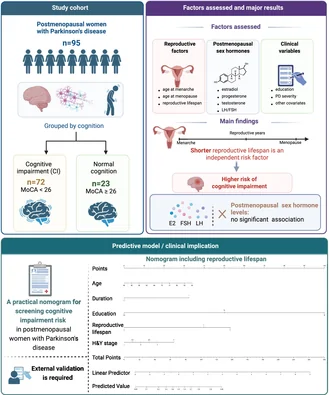

## 1 Introduction

Parkinson's disease (PD) is the second most common neurodegenerative disorder worldwide, affecting approximately 1% of individuals over 60 years of age [1, 2]. Its clinical presentation includes cardinal motor symptoms such as tremor, rigidity, and bradykinesia, as well as a broad spectrum of non‐motor manifestations, such as cognitive impairment, sleep disturbances, autonomic dysfunction, and rapid eye movement sleep behavior disorder [3, 4, 5]. Current PD therapies primarily alleviate symptoms but do not modify the underlying disease course, as the pathogenesis of PD remains insufficiently understood [6, 7]. Consequently, the identification of risk and protective factors is crucial for the development of disease‐modifying interventions.

Beyond established contributors such as aging, genetics, environmental exposures and immune function, biological sex has been increasingly recognized as an important determinant in PD susceptibility and clinical expression [5]. Sex‐related differences are consistently observed, with men exhibiting a higher prevalence of PD, more severe motor symptoms and a faster rate of disease progression than women [8].

The relative protection observed in women has led to the hypothesis that female‐specific biological factors, particularly sex hormones, may contribute to these differences. Estrogen has been the most extensively investigated sex hormone in this context [5, 9, 10, 11, 12, 13, 14]. As a major gonadal steroid hormone, it is believed to play a key role in sex‐related disparities in PD [15]. Preclinical studies have shown that estrogen exerts neuroprotective effects on dopaminergic neurons and supports dopaminergic transmission [16, 17, 18, 19]. Clinical evidence further indicates that postmenopausal women with PD (PWPD) exhibit lower oestradiol and higher testosterone levels than age‐matched postmenopausal controls, including healthy individuals and those with non‐neurodegenerative conditions. Moreover, residual oestradiol production has been associated with milder motor impairment and reduced dopaminergic medication requirements, providing direct in vivo evidence linking sex hormones to PD pathophysiology [20].

Cumulative estrogen exposure across the reproductive lifespan has also been implicated in PD onset and progression. Early or late menarche, higher parity, and surgically induced menopause have been associated with increased PD risk in women [21], whereas longer reproductive lifespan (≥ 40 years) is associated with substantially lower risk compared with a shorter reproductive lifespan (< 30 years) [22]. In addition, shorter reproductive lifespan has been linked to faster motor progression in women with PD [15], and motor symptom severity has been negatively correlated with age at menopause, reproductive lifespan, and duration of endogenous estrogen exposure [23].

Cognitive impairment is among the most common and disabling non‐motor symptoms of PD [24, 25]. Its presence and severity are associated with increased morbidity, reduced life expectancy, significant caregiver burden and higher rates of institutionalization [26]. Prior research in non‐PD populations has identified associations between reproductive factors and cognitive outcomes. Later age at menarche has been associated with poorer cognitive performance [27], whereas later age at menopause and longer reproductive lifespan have been linked to better cognitive function and slower cognitive decline [28]. A study in postmenopausal Hispanic/Latina women reported that longer reproductive lifespan was related to improved verbal learning and processing speed [29]. In hypertensive women, cognitive impairment was positively correlated with age at menarche and negatively correlated with both age at menopause and reproductive lifespan [30]. These findings collectively suggest that cumulative exposure to endogenous estrogen may confer neurocognitive benefits. In PD, however, the relationship between reproductive factors and cognitive function remains unclear. Only limited research has addressed this question. Higher follicle‐stimulating hormone levels have been associated with poorer cognitive performance and with cerebrospinal fluid markers of amyloid pathology and neuronal loss in PWPD [20]. No studies have specifically examined the relationship between premenopausal cumulative sex hormone exposure and cognitive function in postmenopausal patients with PD. Thus, potential associations between reproductive factors and cognitive dysfunction in PD remain insufficiently understood.

The present study aimed to systematically evaluate the associations of reproductive factors and postmenopausal sex hormones with cognitive impairment in PWPD, with a particular focus on distinguishing cumulative premenopausal exposure from postmenopausal residual hormone levels. Furthermore, by identifying independent risk factors, we sought to develop a practical nomogram‐based predictive model to facilitate early identification and risk stratification of cognitive decline in this population.

## 2 Methods

### 2.1 Participants

This was a cross‐sectional observational study. Participants were recruited from the PD‐specialized outpatient clinic of the Aerospace Center Hospital and the Department of Neurology at the First Affiliated Hospital of Guangzhou Medical University from January 2023 to December 2024. The inclusion criteria were as follows: (1) diagnosis of idiopathic PD according to the Movement Disorder Society (MDS) clinical diagnostic criteria were enrolled [31]; (2) postmenopausal female status, defined as amenorrhea for ≥ 12 consecutive months. The exclusion criteria were: (1) parkinsonism‐plus syndromes (e.g., multiple system atrophy, progressive supranuclear palsy, dementia with Lewy bodies); (2) secondary parkinsonism (e.g., vascular, toxic, traumatic, or metabolic); (3) significant psychiatric disorders or severe organic diseases; (4) disorders of consciousness, or current use of thyroid hormones, estrogens, or progesterone supplements; (5) inability to cooperate with scale assessment.

### 2.2 Clinical Assessments

Cognitive function was evaluated using the Montreal Cognitive Assessment (MoCA) [32] and the Mini‐Mental State Examination (MMSE) [33]. Consistent with previously published procedures [34, 35], participants were classified into a cognitive impairment group (PD‐CI; MoCA < 26) and a non‐cognitive impairment group (PD‐NCI; MoCA ≥ 26). Mood symptoms were assessed using the Hamilton Anxiety Rating Scale (HAM‐A) [36] and the Hamilton Depression Rating Scale (HAM‐D) [37]. Motor symptoms were evaluated using the Movement Disorder Society‐Unified PD Rating Scale, Part III (MDS‐UPDRS‐III) [38]. Disease severity was staged according to the Hoehn and Yahr stage (H&Y stage) [39]. All assessments were performed during the ‘on’ medication phase.

### 2.3 General Demographic Characteristics

Demographic and clinical information was collected from all enrolled participants. Demographic variables included age, age at PD onset, height and weight. Clinical characteristics included disease duration, history of thyroid disease, motor phenotype, levodopa equivalent daily dose (LEDD) and the presence of wearing‐off phenomena. LEDD was calculated according to established conversion methods [40].

### 2.4 Assessment of Reproductive Factors

Reproductive factors were obtained through structured self‐reported questionnaires. The information collected included age at menarche and menopause, parity, menstrual characteristics (cycle length and dysmenorrhoea), premenstrual syndrome, history of gynecological surgery and the use of estrogen replacement therapy or oral contraceptives. Reproductive lifespan was defined as the interval between age at menarche and age at menopause. Consistent with prior studies, reproductive variables were categorized as follows: age at menarche (≤ 12 years, 13–14 years and ≥ 15 years) [41]; age at menopause (≤ 44 years, 45–49 years, 50–54 years and ≥ 55 years) [41, 42]; reproductive lifespan (≤ 32 years, 33–38 years and ≥ 39 years) [21]; parity (nulliparous, 1–2 children and ≥ 3 children) [41, 43]; and menstrual cycle length (≤ 24 days, 25–31 days and ≥ 32 days) [21].

### 2.5 Laboratory Measurements

Fasting venous blood samples were collected from all participants between 08:00 and 10:00. An 8 mL blood sample was drawn from the antecubital vein and centrifuged to obtain serum. Serum levels of luteinizing hormone (LH), follicle‐stimulating hormone (FSH), prolactin (PRL), progesterone (P), oestradiol (E2), and total testosterone (TT) were measured using chemiluminescence immunoassay (CLIA) on a Beckman Coulter Dxi 800 automated immunoassay system (Beckman Coulter Inc., Brea, CA, USA), with corresponding manufacturer‐provided reagent kits. All samples were assayed in duplicate, and the mean values were used for statistical analysis.

### 2.6 Statistical Analysis

All statistical analyses were performed using SPSS Statistics version 25.0 (SPSS Inc., Chicago, IL, USA) and R version 4.5.2. Categorical variables are presented as counts and percentages, while continuous variables are expressed as mean ± standard deviation. Between‐group comparisons were conducted using the independent‐samples *t*‐test for normally distributed continuous variables or the Mann–Whitney *U* test for non‐normally distributed variables. The chi‐square test or Fisher's exact test was used for categorical variables, as appropriate. Univariate and multivariate logistic regression analyses were employed to identify potential risk factors for cognitive impairment in PD. Variables with a *p* ≤ 0.05 in the univariate analysis were entered into the multivariate logistic regression model to construct a predictive model. To evaluate the specific impact of reproductive lifespan on cognitive impairment, weighted binary logistic regression models were constructed with two adjustment strategies: Model 1 included no covariate adjustments, and Model 2 was additionally adjusted for covariates identified as clinically relevant and statistically significant. Multicollinearity was assessed using the variance inflation factor (VIF), and all included variables had VIF values < 5, indicating no significant multicollinearity. Sensitivity analyses were conducted to test the robustness of the findings. Subgroup analyses were stratified by age, disease duration, education, H&Y stage, MDS‐UPDRS‐III, HAM‐A, and HAM‐D, with each model adjusting for all other stratification variables except the one under evaluation. Interaction *p* < 0.05 were considered statistically significant. Finally, a nomogram was developed incorporating variables with *p* < 0.05 in the multivariate logistic analysis alongside other clinically important parameters. Model performance was evaluated by discrimination (AUC), calibration (calibration curves with 1000 bootstrap resamples), and clinical utility (decision curve analysis, DCA), with comparisons made against models comprising conventional clinical parameters alone.

## 3 Results

### 3.1 Clinical Characteristics

A total of 95 PWPD were enrolled in the study, including 72 PD‐CI and 23 PD‐NCI patients. As shown in Table 1, the two groups were comparable in age, age at onset, disease duration, body mass index (BMI), HAM‐D, HAM‐A, LEDD, and motor phenotype (all *p* > 0.05). The PD‐CI group had shorter education years, lower MMSE and MoCA scores as well as advanced H&Y stages and higher MDS‐UPDRS‐III scores relative to the PD‐NCI group (all *p* < 0.05).

**TABLE 1:** Comparison of demographic and clinical characteristics, reproductive factors and postmenopausal sex hormone levels between the PD‐CI and PD‐NCI groups.

Characteristic	Total (*n* = 95)	PD‐CI (*n* = 72)	PD‐NCI (*n* = 23)	*p*
Age, years	68.06 ± 8.88	68.97 ± 8.79	65.22 ± 8.74	0.078
Onset age, years	63.06 ± 9.52	63.88 ± 9.69	60.48 ± 8.66	0.136
Disease duration, years	5.00 ± 3.94	5.08 ± 4.00	4.74 ± 3.79	0.717
BMI	23.08 ± 3.24	23.20 ± 3.28	22.71 ± 3.17	0.527
Education	9.73 ± 2.46	9.08 ± 2.47	11.74 ± 0.86	< 0.001
MMSE	24.65 ± 5.17	23.58 ± 5.37	28.00 ± 2.43	< 0.001
MOCA	19.97 ± 6.18	17.64 ± 5.25	27.26 ± 1.01	< 0.001
HAM‐A	9.23 ± 6.96	10.00 ± 7.23	6.83 ± 5.50	0.056
HAM‐D	10.13 ± 7.68	10.69 ± 7.81	8.35 ± 7.10	0.203
H&Y stage	2.35 ± 0.80	2.44 ± 0.76	2.07 ± 0.86	0.047
MDS‐UPDRS‐III	35.56 ± 17.19	37.58 ± 16.75	29.22 ± 17.37	0.041
LEDD, mg/day	468.06 ± 252.73^a^	479.02 ± 260.26^b^	436.13 ± 231.83	0.486
Motor phenotype				0.375
Tremor‐dominant subtype	51 (53.7)	41 (56.9)	10 (43.5)	
Non‐tremor subtype	44 (46.3)	31 (43.1)	13 (56.5)	
Wearing‐off				0.814
No	60 (63.2)	45 (62.5)	15 (65.2)	
Yes	35 (36.8)	27 (37.5)	8 (56.9)	
History of thyroid diseases				0.472
No	84 (88.4)	63 (87.5)	21 (91.3)	
Yes	11 (11.6)	9 (12.5)	2 (8.7)	
Menarche age				0.282
≤ 12 years	11 (11.6)	7 (9.7)	4 (17.4)	
13–14 years	29 (30.5)	20 (27.8)	9 (39.1)	
≥ 15 years	55 (57.9)	45 (62.5)	10 (43.5)	
Menopause age				0.417
< 45 years	10 (10.5)	8 (11.1)	2 (8.7)	
45–49 years	34 (35.8)	28 (38.9)	6 (26.1)	
50–54 years	36 (37.9)	27 (37.5)	9 (39.1)	
≥ 55 years	15 (15.8)	9 (12.5)	6 (26.1)	
Reproductive lifespan				< 0.001^c^, ^d^
≤ 32 years	30 (31.6)	27 (37.5)	3 (13)	
33–38 years	41 (43.2)	34 (47.2)	7 (30.4)	
≥ 39 years	24 (25.3)	11 (15.3)	13 (56.5)	
Menstrual cycle length				0.340
≤ 24 days	4 (4.2)	2 (2.8)	2 (8.7)	
25–31 days	85 (89.5)	66 (91.7)	19 (82.6)	
≥ 32 days	6 (6.3)	4 (5.6)	2 (8.7)	
Parity	1.98 ± 1.34	2.15 ± 1.38	1.43 ± 1.04	0.024
History of menstrual cramps				0.087
No	57 (60)	47 (65.3)	10 (43.5)	
Yes	38 (40)	25 (34.7)	13 (56.5)	
History of gynecological surgery				0.137
No	34 (35.8)	29 (40.3)	5 (21.7)	
Yes	61 (64.2)	43 (59.7)	18 (78.3)	
History of premenstrual syndrome				0.238
No	77 (81.1)	60 (83.3)	17 (73.9)	
Yes	18 (18.9)	12 (16.7)	6 (26.1)	
History of use of estrogen replacement therapy				0.150
No	89 (93.7)	69 (95.8)	20 (87)	
Yes	6 (6.3)	3 (4.2)	3 (13)	
History of oral contraceptives				1.000
No	73 (76.8)	55 (76.4)	18 (78.3)	
Yes	22 (23.2)	17 (23.6)	5 (21.7)	
LH	31.98 ± 14.69	32.00 ± 15.64	31.90 ± 11.50	0.979
FSH	74.95 ± 26.04	75.45 ± 25.92	73.39 ± 26.95	0.743
PRL	8.76 ± 14.48	8.18 ± 11.13	10.55 ± 22.18	0.498
P	0.43 ± 0.28	0.44 ± 0.28	0.37 ± 0.29	0.298
E2	26.01 ± 25.31	22.53 ± 9.89	36.90 ± 47.52	0.164
TT	33.04 ± 15.55	33.41 ± 15.29	31.85 ± 16.65	0.677

*Note:* Data are presented as *n* (%) or mean ± standard deviation.

Abbreviations: BMI, body mass index; E2, oestradiol; FSH, follicle‐stimulating hormone; H&Y stage, Hoehn and Yahr stage; HAM‐A, Hamilton Anxiety Rating Scale; HAM‐D, Hamilton Depression Rating Scale; LEDD, the levodopa equivalent daily dose; LH, luteinizing hormone; MDS‐UPDRS‐III, Movement Disorder Society Unified Parkinson's Disease Rating Scale, Part III; MMSE, the Mini‐Mental State Examination; MoCA, the Montreal Cognitive Assessment; P, progesterone; PD, Parkinson's disease; PD‐CI, postmenopausal PD patients with cognitive impairment; PD‐NCI, postmenopausal PD patients without cognitive impairment; PRL, prolactin; TT, total testosterone.

^a^ *n* = 90.

^b^ *n* = 67.

^c^ Significant difference between the ≤ 32‐year and ≥ 39‐year groups.

^d^ Significant difference between the 33–38‐year and ≥ 39‐year groups.

### 3.2 Comparison of Reproductive Factors and Laboratory Indices Between the PD‐CI and PD‐NCI Groups

Compared with the PD‐NCI group, the PD‐CI group had a higher number of children and a shorter reproductive lifespan with a significantly lower proportion in the ≥ 39‐year category and higher proportions in the shorter categories (both *p* < 0.05). No intergroup differences were observed in age at menarche, age at menopause, menstrual cycle length, menstrual cramps, history of gynecological surgery, history of premenstrual syndrome or prior use of estrogen replacement therapy or oral contraceptives, or serum concentrations of LH, FSH, PRL, P, E2, and TT (both *p* > 0.05). See Table 1.

### 3.3 Reproductive Factors as Independent Risk Factors for Cognitive Impairment

Multivariate logistic regression analysis was performed to identify risk factors for cognitive impairment in postmenopausal patients with PD, including variables that were significant in univariate logistic regression analysis (Table S1). Model 1 was unadjusted; Model 2 was adjusted for age, disease duration, education, BMI, parity, history of menstrual cramps, H&Y stage and MDS‐UPDRS‐III. Compared with a reproductive lifespan ≥ 39 years, the ≤ 32‐year group showed significantly higher odds of cognitive impairment in both Model 1 (OR = 8.81, *p* = 0.015) and Model 2 (OR = 12.41, *p* = 0.011), as did the 33–38‐year group (Model 1: OR = 5.99, *p* = 0.017; Model 2: OR = 8.42, *p* = 0.008). In addition, higher education was associated with significantly lower odds of cognitive impairment in both Model 1 (OR = 0.43, *p* = 0.001) and Model 2 (OR = 0.38, *p* = 0.001). See Table 2.

**TABLE 2:** Multivariable logistic regression analysis identifying factors associated with cognitive impairment in PWPD.

Characteristic	Model 1		Model 2	
	OR (95% CI)	*p*1	OR (95% CI)	*p*2
Age	—	—	1.06 (0.97–1.17)	0.218
Disease duration	—	—	0.97 (0.81–1.16)	0.704
Education	0.43 (0.25–0.72)	0.001	0.38 (0.21–0.69)	0.001
BMI	—	—	1.13 (0.91–1.40)	0.278
Reproductive lifespan				
≤ 32 years	8.81 (1.52–51.03)	0.015	12.41 (1.77–87.23)	0.011
33–38 years	5.99 (1.38–26.03)	0.017	8.42 (1.72–41.07)	0.008
≥ 39 years	1 (Ref)		1 (Ref)	
*p* for trend		0.012		0.008
Parity	0.88 (0.47–1.68)	0.705	0.54 (0.22–1.34)	0.184
History of menstrual cramps				
Yes	—	—	0.62 (0.17–2.29)	0.475
No	—	—	1 (Ref)	
H&Y stage	1.51 (0.43–5.27)	0.523	1.53 (0.44–5.31)	0.507
MDS‐UPDRS‐III	0.98 (0.92–1.05)	0.574	1.00 (0.93–1.06)	0.887

*Note:* Model 1: unadjusted model. Model 2: adjusted for age, disease duration, education, BMI, parity, history of menstrual cramps, H&Y stage and MDS‐UPDRS‐III.

Abbreviations: BMI, body mass index; H&Y stage, Hoehn and Yahr stage; MDS‐UPDRS‐III, Movement Disorder Society Unified Parkinson's Disease Rating Scale, Part III; PD, Parkinson's disease; PWPD, postmenopausal women with PD.

### 3.4 Sensitivity Analyses Assessing the Robustness of the Association Between Reproductive Lifespan and Cognitive Impairment

In the first analysis, cognitive impairment was defined using education‐stratified MoCA cutoff scores: ≤ 13 for illiterate participants, ≤ 19 for those with 1–6 years of schooling, and ≤ 24 for those with ≥ 7 years of education [44]. Using reproductive lifespan ≥ 39 years as the reference, individuals with ≤ 32 years and 33–38 years had odds ratios (ORs) for cognitive impairment of 4.61 (95% CI, 1.29–16.47) and 4.59 (95% CI, 1.43–14.79), respectively, in Model 1; the corresponding adjusted ORs in Model 2 were 5.37 (95% CI, 1.44–20.02) and 4.74 (95% CI, 1.44–15.64) (both *p* < 0.05). Moreover, across increasing quartiles of reproductive lifespan, the prevalence of cognitive impairment showed a significant decreasing trend in both models (Model 1: *p* for trend = 0.021; Model 2: *p* for trend = 0.013). See Table 3.

**TABLE 3:** Results of sensitivity analyses evaluating the association between the reproductive lifespan and cognitive impairment in PWPD.

Characteristic	Model 1 (unadjusted)	Model 2 (multivariable‐adjusted)		
	OR (95% CI)	*p*1	OR (95% CI)	*p*2
**Sensitivity Analysis 1** ***(Cognitive impairment defined by education‐adjusted MoCA cutoffs)***				
Reproductive lifespan				
≤ 32 years	4.61 (1.29–16.47)	0.019	5.37 (1.44–20.02)	0.012
33–38 years	4.59 (1.43–14.79)	0.011	4.74 (1.44–15.64)	0.011
≥ 39 years	1 (Ref)		1 (Ref)	
*p* for trend		0.021		0.013
**Sensitivity Analysis 2** ***(Excluding participants with MMSE < 20)***				
Reproductive lifespan				
≤ 32 years	7.21 (1.35–38.36)	0.021	12.71 (1.77–91.46)	0.012
33–38 years	6.65 (1.62–27.24)	0.008	10.74 (1.98–58.22)	0.006
≥ 39 years	1 (Ref)		1 (Ref)	
*p* for trend		0.012		0.008

*Note:* Model 1: Unadjusted model. Model 2: Adjusted for age, disease duration, education, BMI, parity, history of menstrual cramps, H&Y stage and MDS‐UPDRS‐III.

Abbreviations: MMSE, the Mini‐Mental State Examination; MoCA, the Montreal Cognitive Assessment; PD, Parkinson's disease; PWPD, postmenopausal women with PD.

In the second sensitivity analysis, we excluded 14 participants with an MMSE score < 20, who were considered to have possible dementia [45]. Relative to the reference group (reproductive lifespan ≥ 39 years), individuals with ≤ 32 years and 33–38 years had ORs of 7.21 (95% CI, 1.35–38.36) and 6.65 (95% CI, 1.62–27.24), respectively, in Model 1; the corresponding adjusted ORs in Model 2 were 12.71 (95% CI, 1.78–91.46) and 10.74 (95% CI, 1.98–58.22) (both *p* < 0.05). Similarly, a significant decreasing trend in cognitive impairment prevalence was observed across ascending quartiles of reproductive lifespan in both models (Model 1: *p* for trend = 0.012; Model 2: *p* for trend = 0.008). See Table 3.

### 3.5 Subgroup Analyses

Subgroup analyses were performed to evaluate potential effect modifiers of the association between reproductive lifespan and cognitive impairment in PD. As shown in Figure 1, no significant interactions were observed for age, disease duration, HAM‐A, or HAM‐D (all *p* for interactions > 0.05). In contrast, significant interactions were found for education, H&Y stage, and MDS‐UPDRS‐III (all *p* for interactions < 0.05), with the association between reproductive lifespan and cognitive impairment being more pronounced in patients with education ≥ 12 years, H&Y stage ≤ 2.5, and MDS‐UPDRS‐III < 33 (Figure 1).

**FIGURE 1:**
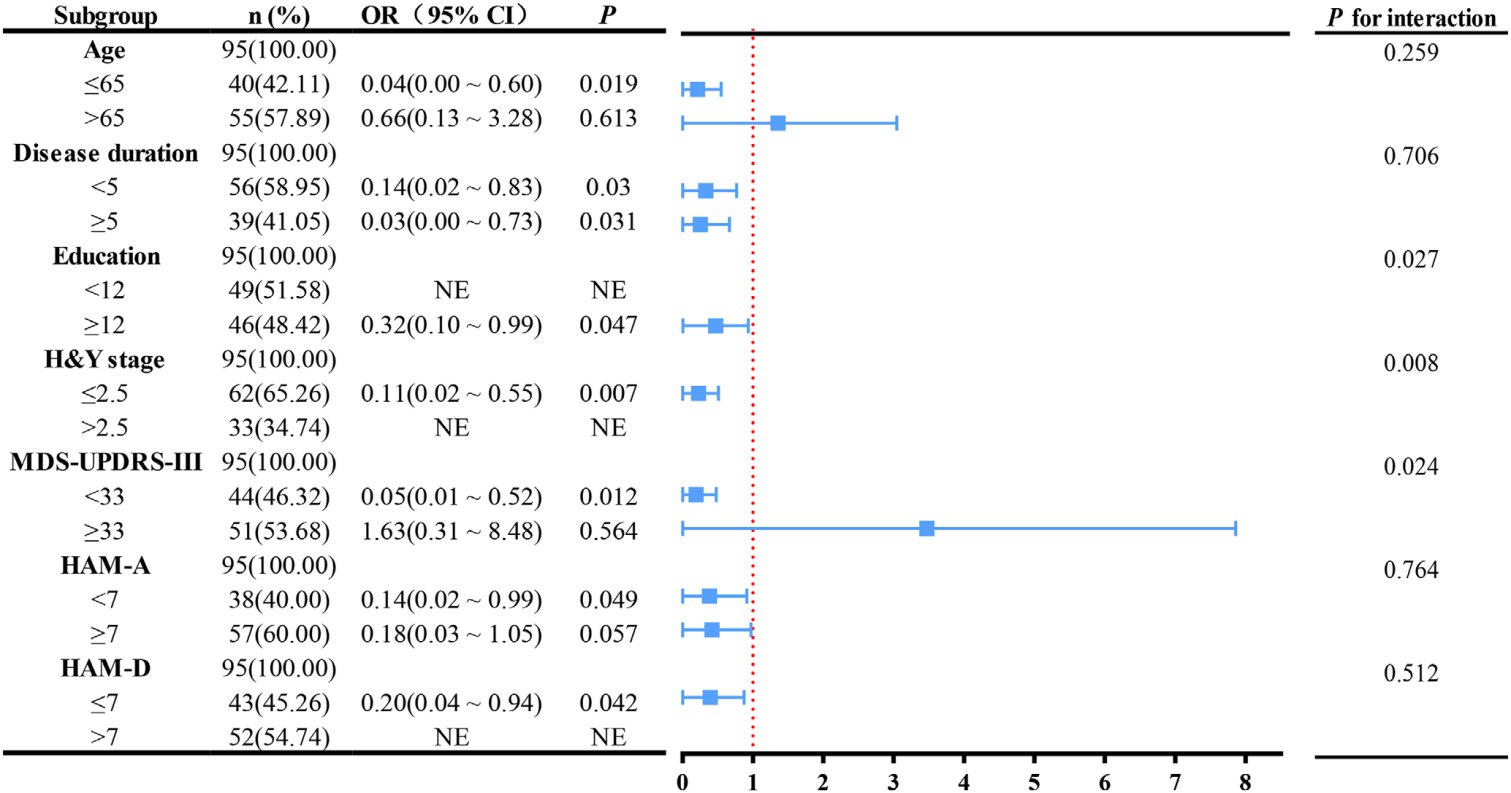
Subgroup analyses of the association between reproductive lifespan and cognitive impairment. Multivariate logistic regression was adjusted for all other stratification variables except the stratified variable. No interactions were significant for age, disease duration, HAM‐A and HAM‐D; significant interactions were observed for education, H&Y stage and MDS‐UPDRS‐III. The association of reproductive lifespan with cognitive impairment was enhanced in participants with education ≥ 12 years, H&Y stage ≤ 2.5, and MDS‐UPDRS‐III < 33. Interaction *p* < 0.05 indicated statistical significance. H&Y stage, Hoehn and Yahr stage; HAM‐A, Hamilton Anxiety Rating Scale; HAM‐D, Hamilton Depression Rating Scale; MDS‐UPDRS‐III, Movement Disorder Society Unified Parkinson's Disease Rating Scale Part III.

### 3.6 Construction and Validation of the Nomogram Model

A nomogram incorporating five predictors (reproductive lifespan, education, age, disease duration, and H&Y stage) was constructed as the full model (Model 1). Although age, disease duration, and H&Y stage were not statistically significant in the multivariate logistic regression analysis, they were retained due to their well‐documented clinical relevance to cognitive decline in PD [46, 47, 48]. The scoring system and total risk scale are presented in Figure 2.

**FIGURE 2:**
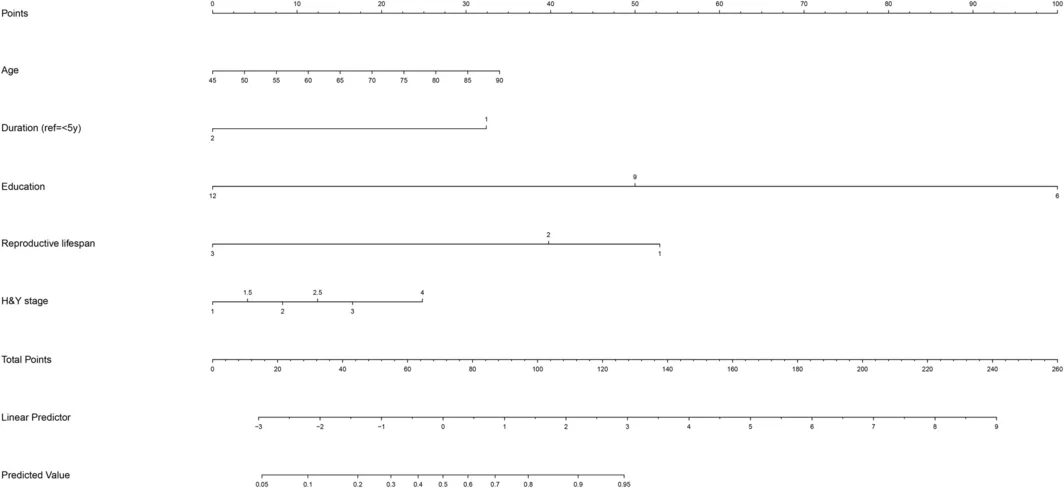
Nomogram for predicting cognitive impairment in postmenopausal women with PD. A nomogram (Model 1) was established incorporating five predictors: reproductive lifespan, education, age, disease duration, and H&Y stage. H&Y stage, Hoehn and Yahr stage; PD, Parkinson's Disease.

Four prediction models were evaluated using ROC curves (Figure 3A), calibration curves (Figure 3B), and DCA (Figure 3C): the full model (Model 1), a conventional clinical model (Model 2: age, education, disease duration, and H&Y stage), a model with only the significant independent factors (Model 3: education and reproductive lifespan), and a model with reproductive lifespan alone (Model 4). Model 1 achieved an AUC of 0.903, with a calibration intercept of −0.024, slope of 1.093, Brier score of 0.109, and Hosmer–Lemeshow (HL) *p* = 0.170. Compared with Model 2 (AUC = 0.842, Brier = 0.132, HL *p* = 0.555), Model 1 showed substantially higher AUC and lower Brier score, indicating that the addition of reproductive lifespan markedly improved the predictive performance of conventional clinical factors. Model 4 (AUC = 0.732, Brier = 0.152, HL *p* < 0.001) showed poor calibration, whereas Model 3 (AUC = 0.864, Brier = 0.122, HL *p* = 0.861) exhibited good discrimination with excellent calibration. In DCA, Model 1 consistently provided the highest net benefit across threshold probabilities of 0.25–0.50, outperforming the other models. Overall, Model 1 demonstrated favorable discrimination, acceptable calibration, and the greatest net clinical benefit across the clinically relevant threshold spectrum. Detailed calibration and DCA parameters for all models are available in Tables S1 and S3.

**FIGURE 3:**
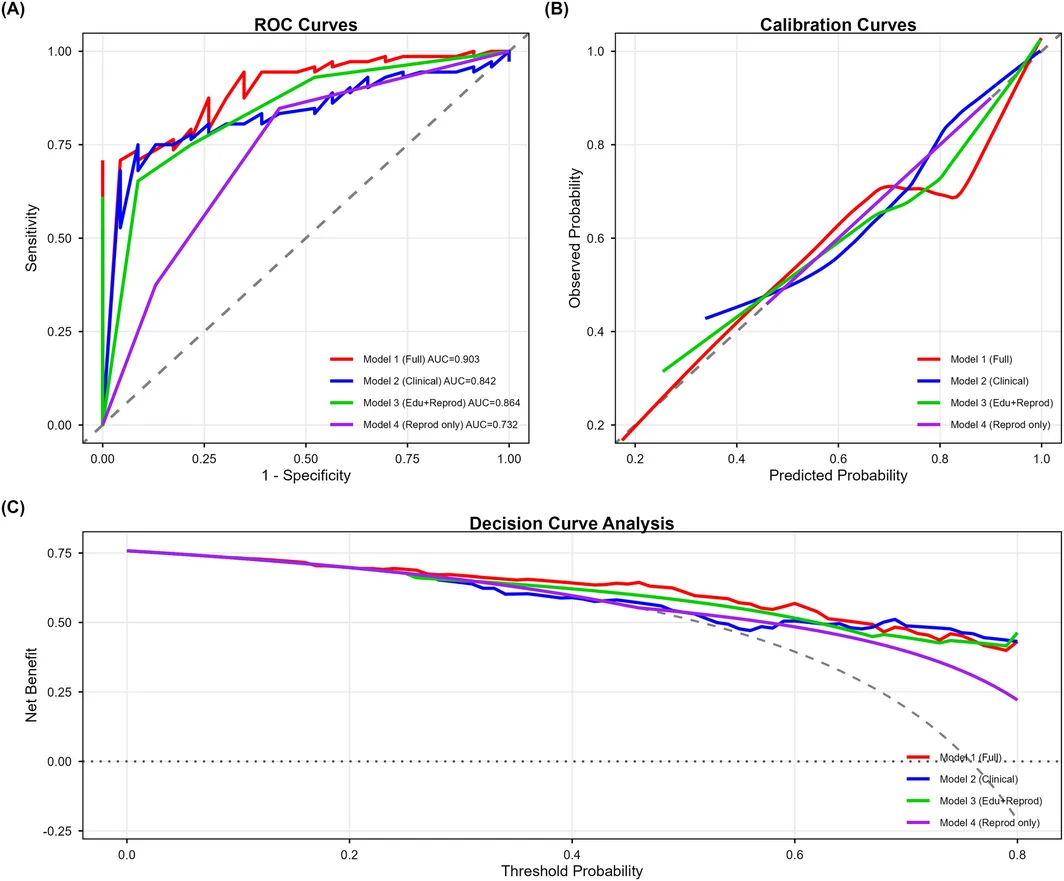
Performance comparison of four predictive models for cognitive impairment in PD. Discrimination, calibration and clinical utility of the four models were evaluated using receiver operating characteristic (ROC) curves (A), 1000‐bootstrap calibration curves (B), and decision curve analysis (DCA) (C). Definitions of four predictive models: Model 1 (full): Reproductive lifespan, education, age, disease duration, and H&Y stage; Model 2 (clinical): Education, age, disease duration, and H&Y stage; Model 3 (Edu + Reprod): Education and reproductive lifespan; Model 4 (Reprod only): Reproductive lifespan alone. Model 1 showed the highest AUC (0.903), acceptable calibration, and optimal net clinical benefit compared with the other three models. AUC, area under the ROC curve; DCA, decision curve analysis; H&Y stage, Hoehn and Yahr stage; PD, Parkinson's disease; ROC, receiver operating characteristic.

## 4 Discussion

Cognitive impairment in PD has gained increasing research attention in recent decades. In this study, we examined the associations of reproductive factors and circulating sex hormones with cognitive impairment in PWPD. Our results showed that shorter reproductive lifespan was independently associated with cognitive impairment, particularly in patients with milder disease severity, whereas postmenopausal sex hormone levels showed no such association. Additionally, we developed a predictive nomogram integrating reproductive lifespan and routine clinical and demographic parameters, and internal validation verified its favorable discriminatory ability and clinical applicability.

The association between reproductive factors and cognitive performance has been well documented in the general population across diverse populations [49, 50, 51, 52]. Prior studies have demonstrated that reproductive factors, including age at menarche and menopause, parity and reproductive lifespan, can influence cognitive performance. The majority of evidence indicates that earlier menarche, later menopause, and a longer reproductive lifespan are correlated with better cognitive performance and a lower risk of cognitive impairment, while higher parity is associated with poorer cognitive function [29, 30, 50, 53]. These associations are broadly attributed to the neuroprotective effects of sex hormones, particularly estrogen.

In PD, although shorter reproductive lifespan, earlier menopause, and higher parity have been associated with increased risk of PD and accelerated disease progression [15, 22, 23, 54], their relationship with cognitive function remains largely unexplored. Schirò et al. [55] reported that earlier menarche was associated with better cognitive performance in PWPD. The present study identified reproductive lifespan as an independent risk factor significantly associated with cognitive impairment, with shorter reproductive lifespan correlating with a higher prevalence of cognitive deficits. The robustness of this association was confirmed by sensitivity analyses that either excluded patients with dementia or redefined cognitive impairment using education‐adjusted MoCA cutoffs.

Subgroup analyses demonstrated that the association between reproductive lifespan and cognitive impairment remained generally stable across different strata, except education, H&Y stage, and MDS‐UPDRS III, with the association being more pronounced in patients with education ≥ 12 years, H&Y stage ≤ 2.5, or MDS‐UPDRS III < 33. These findings suggest that the association was more pronounced in patients with milder disease severity. This may be attributed to the fact that in advanced PD, the irreversible loss of dopaminergic neurons creates a massive pathological burden [56], which largely overwhelms the subtle cumulative advantages conferred by a longer reproductive lifespan. The interaction with education suggests that in patients with lower education, the strong adverse effect of limited education likely masks the modest protective effect of reproductive lifespan, whereas the protective effect becomes more detectable in those with higher education, potentially reflecting an interplay between cognitive reserve [57] and estrogen‐mediated neuroprotection [58].

Few studies have evaluated postmenopausal endogenous hormone levels or their relevance to PD‐related outcomes. Bovenzi et al. [20] found that PWPD had lower E2 and higher TT levels than postmenopausal healthy controls, and that FSH levels were correlated with poorer cognitive performance, suggesting that circulating postmenopausal sex hormones may influence cognitive outcomes in women with PD. In contrast to their findings, we observed no significant differences in postmenopausal sex hormone levels between PD‐CI and PD‐NCI. The discrepancy may be attributable to differences in ethnicity, sample characteristics, or inclusion criteria.

Sex steroid hormones influence neural circuits involved in attention, memory and executive function [29, 59]. Estrogens, in particular, have been shown to modulate both the structure and function of brain regions such as the hippocampus and prefrontal cortex, regions rich in estrogen receptors and critical for learning, memory and executive processes [60]. Furthermore, estrogens also have anti‐inflammatory, neurotrophic and neuroprotective effects and can upregulate nigrostriatal dopaminergic activity [21]. These mechanisms support the observed relationship between reproductive lifespan and cognitive impairment in PWPD. Collectively, our findings suggest that endogenous sex hormones secreted during the reproductive period may confer long‐term neuroprotective effects on cognitive function in PD, whereas postmenopausal residual hormone levels may not provide comparable protection. This interpretation, however, should be interpreted with caution, given the absence of premenopausal hormone measurements and the lack of longitudinal data in this postmenopausal cohort.

We developed a nomogram (Model 1) by integrating two independent risk factors identified by multivariate logistic regression—reproductive lifespan and education—with three clinically relevant variables (age, disease duration, and H&Y stage). The model exhibited good discrimination (AUC = 0.903), satisfactory calibration, and favorable net clinical benefit over a broad threshold range of 0.10–0.80.

Compared with the conventional clinical model (Model 2: age, education, disease duration, and H&Y stage), Model 1 showed a substantially higher AUC (0.903 vs. 0.842), confirming that adding reproductive lifespan significantly improves predictive performance. This finding underscores the incremental value of reproductive lifespan as a sex‐specific predictor beyond traditional risk factors in PWPD. Notably, a simplified model comprising only education and reproductive lifespan (Model 3) achieved an AUC of 0.864 with good calibration, comparable to Model 2, suggesting that these two variables alone may offer a practical brief screening tool in time‐constrained clinical settings. In contrast, reproductive lifespan alone (Model 4) performed poorly (AUC = 0.732, poor calibration), indicating that it should not be used in isolation for risk prediction.

Interestingly, the nomogram unexpectedly showed that shorter disease duration was associated with higher cognitive risk—contrary to conventional expectation. This counterintuitive finding may be largely attributable to survival and loss‐to‐follow‐up biases, as patients with rapid progression and early cognitive decline tend to die prematurely from complications or drop out due to severe disability, leaving the long‐duration group selectively depleted of high‐risk cases. However, we cannot exclude the alternative possibility that this inverse association reflects inherent disease subtype differences, with early‐onset or rapidly progressive forms conferring higher cognitive risk regardless of disease duration.

Overall, our nomogram offers a novel approach by integrating reproductive lifespan with routine clinical indicators for cognitive impairment screening in PWPD. However, external validation in independent, multicenter prospective cohorts is warranted before clinical implementation.

This study has several limitations. First, the relatively limited sample size and subgroup imbalance between PD‐CI and PD‐NCI may result in unstable regression model estimates. Second, reproductive factors were retrospectively collected via self‐report, which may have introduced recall bias, particularly among patients with cognitive impairment. Third, the observational design may be subject to residual confounding from unmeasured variables. Although we adjusted for several important covariates—including age, disease duration, BMI, education, and disease severity—reproductive lifespan may not exclusively reflect a direct hormonal effect. It could also serve as a proxy for broader biological, lifestyle, or social determinants—such as nutritional status, dietary habits, healthcare access, comorbidities, and socioeconomic conditions, which may influence both reproductive development and late‐life cognitive outcomes. Therefore, residual confounding by these unmeasured or imperfectly measured factors cannot be fully excluded.

Despite these limitations, this study has several notable strengths. It specifically addresses the under‐explored association between reproductive factors and PD‐related cognitive impairment and provides a preliminary predictive model tailored to PWPD. Moreover, the simultaneous evaluation of both reproductive factors and postmenopausal sex hormone levels offers an integrative perspective on how hormone exposure before and after menopause may influence cognitive outcomes in PD. Future studies should aim to further validate and enhance the accuracy of the prediction model in large‐scale, multicenter prospective cohort.

In conclusion, we found that reproductive lifespan was independently associated with cognitive impairment in PWPD, with a shorter reproductive lifespan linked to a higher prevalence of cognitive impairment, and this association was more pronounced in patients with milder disease severity. In contrast, postmenopausal sex hormone levels showed no association with cognitive status. Furthermore, we also developed a nomogram incorporating reproductive lifespan and routine clinical parameters, which demonstrated good predictive discrimination and net benefit, offering a potential practical tool for cognitive risk stratification in this population, pending external validation before clinical implementation.

## Author Contributions

Wen‐Zheng Hu and Qin Wang drafted and revised the manuscript, conducted clinical assessments, and collected and analyzed the data. Ling Tang, Jia‐Qi Wang, Di Hu and Teng‐Teng Wu conducted clinical assessments and contributed to data collection. Zhi‐Hong Shi and Yue Huang contributed to data collection. Yi Ren contributed to the manuscript revision. Zhi‐Rong Wan and Ping‐Yi Xu conceived and designed the study, oversaw patient recruitment, performed critical manuscript revisions and approved the final version. All authors contributed to the article and approved the submitted manuscript.

## Funding

This work was supported by the National Natural Science Foundation of China (grant number U25A2059), the Science and Technology Projects in Guangzhou (grant number 2024B03J0904), the Natural Science Foundation of Guangdong Province of China (grant numbers 2023A1515011897, 2023A1515140070 and 2025A1515011146), and the Project of Aerospace Center Hospital (grant number YN202403).

## Ethics Statement

This study involving human participants was reviewed and approved by the Medical Ethics Committee of the Aerospace Center Hospital (Approval No. 20190614‐SF‐06) and the First Affiliated Hospital of Guangzhou Medical University (Approval No. ES‐2024‐216‐01).

## Consent

All participants provided written informed consent prior to enrolment.

## Conflicts of Interest

The authors declare that the research was conducted in the absence of any commercial or financial relationships that could be perceived as potential conflicts of interest. The authors report no competing interests.

## Supporting information


**Table S1:** Univariate logistic regression analysis of factors associated with cognitive impairment in postmenopausal women with PD.
**Table S2:** Performance metrics of the four prediction models: discrimination and calibration.
**Table S3:** Net benefit estimates from decision curve analysis at selected threshold probabilities.

## Data Availability

The data that support the findings of this study are available from the corresponding author upon reasonable request.
