# Supplementary material for: Association of Reproductive Factors and Postmenopausal Sex Hormones With Cognitive Impairment in Postmenopausal Women With Parkinson's Disease
Source: CNS Neurosci Ther. 2026 Oct 8;32(10):e71182. doi: 10.1002/cns.71182 (PMC13647490; doi:10.1002/cns.71182)
Supplement: Supplementary file 1 [file CNS-32-e71182-s001.docx]

**Supplementary Table S1.** Univariate logistic regression analysis of factors associated with cognitive impairment in postmenopausal women with PD.

| **Variable** | **OR (95%CI)** | ***p*** |
| --- | --- | --- |
| Age, years | 1.05 (0.99-1.11) | 0.082 |
| Onset age, years | 1.04 (0.99-1.09) | 0.138 |
| Disease duration, years | 1.02 (0.91-1.16) | 0.714 |
| BMI | 1.05 (0.91-1.22) | 0.523 |
| Education | 0.44 (0.27-0.70) | **0.001** |
| MMSE | 0.61 (0.46-0.81) | **0.001** |
| HAM-A | 1.08 (1.00-1.18) | 0.062 |
| HAM-D | 1.04 (0.98-1.12) | 0.204 |
| H&Y stage | 1.86 (1.00-3.44) | **0.050** |
| MDS-UPDRS-III | 1.03 (1.00-1.07) | **0.045** |
| LEDD, mg/day ^a^ | 1.00 (1.00-1.00) | 0.481 |
| Motor phenotype |  |  |
| Tremor-dominant subtype | Ref |  |
| Non-tremor subtype | 0.58 (0.23-1.50) | 0.262 |
| Wearing-off |  |  |
| No | Ref |  |
| Yes | 1.13 (0.42-3.00) | 0.814 |
| History of thyroid diseases |  |  |
| No | Ref |  |
| Yes | 1.50 (0.30-7.50) | 0.622 |
| Menarche age |  |  |
| ≤12 years | Ref |  |
| 13–14 years | 1.27 (0.30- 5.46) | 0.748 |
| ≥15 years | 2.57 (0.63- 10.50) | 0.188 |
| Menopause age |  |  |
| <45 years | Ref |  |
| 45-49 years | 1.17 (0.20-6.94) | 0.865 |
| 50-54 years | 0.75 (0.13-4.20) | 0.744 |
| ≥55 years | 0.38 (0.06-2.41) | 0.302 |
| Reproductive lifespan |  |  |
| ≤32 years | 10.64 (2.53-44.80) | **0.001** |
| 33–38 years | 5.74 (1.83-18.00) | **0.001** |
| ≥39 years | Ref |  |
| Menstrual cycle length |  |  |
| ≤24 days | Ref |  |
| 25–31 days | 3.47 (0.46-26.33) | 0.228 |
| ≥32 days | 2.00 (0.15-26.73) | 0.600 |
| Parity | 1.82 (1.06-3.15) | **0.031** |
| History of menstrual cramps |  |  |
| No | Ref |  |
| Yes | 0.41 (0.16-1.07) | 0.067 |
| History of gynaecological surgery |  |  |
| No | Ref |  |
| Yes | 0.41 (0.14-1.23) | 0.113 |
| History of premenstrual syndrome |  |  |
| No | Ref |  |
| Yes | 0.57 (0.19-1.73) | 0.319 |
| History of use of estrogen replacement therapy |  |  |
| No | Ref |  |
| Yes | 0.29 (0.05-1.55) | 0.148 |
| History of oral contraceptives |  |  |
| No | Ref |  |
| Yes | 1.11 (0.36-3.45) | 0.853 |
| LH | 1.00 (0.97-1.03) | 0.978 |
| FSH | 1.00 (0.99-1.02) | 0.740 |
| PRL | 0.99 (0.96-1.02) | 0.505 |
| P | 2.69 (0.42-17.36) | 0.297 |
| E2 | 0.97 (0.93-1.00) | 0.079 |
| TT | 1.00 (0.98-1.04) | 0.673 |

^a^n = 90.

Abbreviations: PD, Parkinson’s disease; BMI, body mass index; MMSE, the Mini-Mental State Examination; HAM-A, Hamilton Anxiety Rating Scale; HAM-D, Hamilton Depression Rating Scale; H&Y stage, Hoehn and Yahr stage; MDS-UPDRS-III, Movement Disorder Society–Unified Parkinson’s Disease Rating Scale, Part III; LEDD, the levodopa equivalent daily dose; LH, luteinising hormone; FSH, follicle-stimulating hormone; PRL, prolactin; P, progesterone; E2, oestradiol; TT, total testosterone.

**Supplementary Table S2.** Performance metrics of the four prediction models: discrimination and calibration

| **Model** | **AUC (95% CI)** | **Intercept** | **Slope** | **Brier** | **HL *p*** |
| --- | --- | --- | --- | --- | --- |
| Model 1 | 0.903 (0.843–0.964) | -0.024 | 1.093 | 0.109 | 0.170 |
| Model 2 | 0.842 (0.765–0.920) | 0.115 | 0.797 | 0.132 | 0.555 |
| Model 3 | 0.864 (0.794–0.934) | -0.035 | 1.224 | 0.122 | 0.861 |
| Model 4 | 0.732 (0.616–0.849) | 0.164 | 0.875 | 0.152 | <0.001 |

Model 1 (full): reproductive lifespan, education, age, disease duration, and H&Y stage; Model 2 (conventional clinical model): education, age, disease duration, and H&Y stage; Model 3 (significant independent factors only): education and reproductive lifespan; Model 4 (reproductive lifespan only): reproductive lifespan alone. HL, Hosmer–Lemeshow test.

**Supplementary Table S3.** Net benefit estimates from decision curve analysis at selected threshold probabilities

| **Threshold** | **Model 1** | **Model 2** | **Model 3** | **Model 4** |
| --- | --- | --- | --- | --- |
| 0.25 | 0.691 | 0.677 | 0.677 | 0.677 |
| 0.30 | 0.669 | 0.644 | 0.651 | 0.654 |
| 0.35 | 0.653 | 0.602 | 0.637 | 0.628 |
| 0.40 | 0.642 | 0.590 | 0.621 | 0.597 |
| 0.50 | 0.611 | 0.537 | 0.579 | 0.537 |
| Utility Range | 0.10 - 0.80 | 0.43 - 0.80 | 0.32 - 0.80 | 0.46 - 0.80 |

Model 1 (full): reproductive lifespan, education, age, disease duration, and H&Y stage; Model 2 (conventional clinical model): education, age, disease duration, and H&Y stage; Model 3 (significant independent factors only): education and reproductive lifespan; Model 4 (reproductive lifespan only): reproductive lifespan alone.
